# Psoriasis in Childbearing Age: A Real-Life, Retrospective, Single-Center Study on Anti-IL17 and IL-23 Agents

**DOI:** 10.3390/jcm13216401

**Published:** 2024-10-25

**Authors:** Silvia Borriello, Gabriele Roccuzzo, Paolo Dapavo, Nadia Sciamarrelli, Nicole Macagno, Francesco Leo, Pietro Quaglino, Simone Ribero, Luca Mastorino

**Affiliations:** Section of Dermatology, Department of Medical Sciences, University of Turin, 10126 Turin, Italy; silvia.borriello@edu.unito.it (S.B.); paolodapavo@gmail.com (P.D.); nadia.sciamarelli@edu.unito.it (N.S.); nicole.macagno@edu.unito.it (N.M.); francesco.leo144@edu.unito.it (F.L.); pietro.quaglino@unito.it (P.Q.); simone.ribero@unito.it (S.R.); luca.mastorino@unito.it (L.M.)

**Keywords:** psoriasis, biologic therapy, ixekizumab, secukinumab, brodalumab

## Abstract

**Background:** Psoriasis (PSO) involves about 1–3% of the population, and around 75% of women develop PSO before the age of 40. Official guidelines on the treatment of woman with anti-IL17 and anti-IL23 during this potential childbearing time are not currently available. **Objective:** To investigate the effectiveness and safety of biologic treatments in women of childbearing age. **Methods:** At the Dermatology Clinic of the University of Turin, Italy, 172 women aged 20–55 years underwent IL-17 and IL-23 inhibitor therapy for moderate-severe PSO and were followed up to 52 weeks in a real-life, retrospective, single-center study. **Results:** Overall, 40% of patients achieved PASI 100 at week 16, and 72% at week 52. A similar trend was observed for PASI 90 and PASI < 3 with almost 80% and 90% of patients achieving the target at 52 weeks. As for PASI 100, a statistically significant difference (*p* = 0.004) was found at 16 weeks, with 23.84% of patients achieving PASI 100, with IL-17 and 15.70% with the IL-23 inhibitor. No statistically significant differences were found at 28 and 52 weeks. No statistically significant differences in PASIs at any time points were recorded across the different biologic agents. Forty-six patients discontinued treatment. The most reported side effects were headache, nausea, asthenia, injection site pain, and rhinitis. **Conclusions:** This study showed that biologic drugs are effective and safe in women of childbearing age, with negligible side effects.

## 1. Introduction

Psoriasis (PSO) is a chronic inflammatory disease affecting approximately 1–3% of the white population, with similar prevalence rates among men and women [1]. Notably, PSO in women typically develops in two distinct age ranges: 16–22 and 55–60, with around 75% of female cases occurring before age 40, aligning with the childbearing years, and an average diagnosis age of 28 [2,3]. From 65,000 to 107,000 births from women with psoriasis occur each year, and from 9000 to 15,000 births from women with moderate to severe disease [4].

The severity of psoriasis can vary during pregnancy, and up to 23% of women with psoriasis may experience a worsening of the clinical picture [5]. In addition, pregnant patients with psoriasis have been found to have an increased risk of pregnancy-, birth- and post-partum-related diseases such as pre-eclampsia, pregnancy-related hypertension, severe post-partum hemorrhage, preterm delivery, and miscarriage [6]. This could be explained by an increase in and dysregulation of certain inflammatory cytokines and chemokines, such as an increase in tumor necrosis factor (TNF)-alpha. In fact, it has been found that elevated serum or umbilical cord levels of tumor necrosis factor-α, C-reactive protein, and interleukin-6, which correlate with the severity of psoriasis, can lead to preterm delivery and SGA infants [7].

Recent scientific advancements have significantly reshaped our understanding of PSO’s underlying mechanisms, leading to the discovery of novel molecular pathways [8,9]. These insights have facilitated the development of targeted therapies, including biologic treatments, which have demonstrated remarkable efficacy in managing the condition [8,9].

Among the traditional systemic drugs, cyclosporine is the only one approved for severe psoriasis in pregnancy. However, it is not without side effects. In fact, cases of premature labor and low birth weight in newborns have been reported in women with psoriasis treated with cyclosporine [10]. Methotrexate and acitretin are contraindicated in pregnancy due to their teratogenic effects. The FDA classifies them as category X [11]. Acitretin should also be avoided in women of childbearing age or at least stopped three years before possible conception [11]. Treatment with acitretin during pregnancy could lead to severe malformation of the fetus with cardiac, neurological, thymic, and craniofacial defects [12].

When cyclosporine is not indicated due to the patient’s comorbidities or in case of therapeutic failure, biologic drugs can offer an excellent therapeutic alternative. However, studies of the latter in patients of childbearing age and pregnancy are very limited because patients receiving systemic treatment often discontinue treatment during pregnancy and because pregnant women are usually excluded from clinical trials for ethical reasons [13,14]. Indeed, The British Association of Dermatologists guidelines recommend an effective contraceptive method for women of childbearing age undergoing biological therapy and that continuing or discontinuing biological therapy at the time of an established pregnancy should be discussed on a case-by-case basis in pregnant women [15].

The biological therapies currently available for psoriasis are derivatives of human immunoglobulin G (IgG) and are transported across the placenta through neonatal Fc receptors [16]. Monoclonal antibodies are transferred across the placenta more efficiently, as early as the 13th week of gestation. Indeed, in the first trimester of pregnancy, there is not yet sufficient Fc for transport, and biological drugs will cross the placenta mainly by passive diffusion. Thus, the drug concentration that will reach the fetus is insignificant [16]. The monoclonal antibodies have different affinities for Fc receptors. For example, IgG1 (guselkumab, adalimumab, infliximab, risankizumab, secukinumab, ustekinumab, risankizumab, tildrakizumab, and bimekizumab) is the most easily transportable subtype, followed by IgG4 (ixekizumab), IgG3, and IgG2 (brodalumab) [13]. Instead, certolizumab pegol is a pegylated IgG1 human monoclonal antibody with reduced or no placental transfer due to the absence of an Fc receptor [8,17].

According to the EuroGuiDerm guideline on the systemic management of psoriasis vulgaris, certolizumab pegol is recommended as the preferred biologic therapy for women planning pregnancy or already pregnant and requiring systemic treatment (from July 2018) [17]. In addition, certolizumab pegol can be used throughout pregnancy, without the need for an interruption [17].

The other TNF-α inhibitors, such as adalimumab, golimumab, etanercept, and infliximab, can cross the placenta from the second trimester onwards and remain detectable in the newborn for up to a year after delivery, raising concerns about the potential impact on the baby’s immune system [18]. They may be recommended in pregnant women or women who plan to become pregnant and require systemic treatment, but such treatment should be limited to the first and second trimesters of pregnancy [19]. Concerning the use of etanercept during pregnancy, one case reported infants born with VATER syndrome (V: vertebral anomalies; A: anal anomalies; T: tracheal problems; E: esophageal problems; R: radial or renal defects) from a patient with psoriasis and psoriatic arthritis who received etanercept during pregnancy [20]. Administration of live vaccines should be postponed after 6 months in infants exposed to anti-TNF agents during pregnancy [21].

Recently, interleukin 23 and 17 (IL-23/17) inhibitors have emerged as effective and safe options for treating moderate-to-severe psoriasis [22,23]. However, according to the 2020 American College of Rheumatology Guidelines, secukinumab and ustekinumab should be discontinued during pregnancy [24]. Concerning interleukin 23 inhibitors, with regard to the IL-23A inhibitor risankizumab, there are limited data on its use in the treatment of psoriasis in pregnant women; therefore, it is recommended not to use this agent during pregnancy and to use a contraceptive method during treatment and for a minimum of 21 weeks after its completion [25,26]. In addition, it is recommended to avoid pregnancy with guselkumab and tildrakizumab and to use a contraceptive method for at least 12 weeks (guselkumab)/17 weeks (tildrakizumab) after stopping treatment [26,27].

Although some studies suggest that the risk of teratogenic effects from biologic drugs is low, particularly during the first trimester, their effectiveness and safety in this demographic warrant careful evaluation [28,29]. Considering the importance of patient autonomy and disease severity, our research focuseed on exploring the safety and effectiveness of interleukin-17 and -23 inhibitors in women of childbearing age.

## 2. Materials and Methods

This study was a monocentric, retrospective clinical investigation that included all consecutive female patients diagnosed with either psoriasis or psoriatic arthritis (PsA) who received at least one dose of IL-23 or IL-17 inhibitors between January 2017 and June 2022. This study was conducted at the Dermatologic Clinic of the University of Turin, a tertiary referral center specializing in psoriasis management located in northern Italy. This study was carried out in strict adherence with the principles outlined in the Declaration of Helsinki. Ethical approval was obtained from the institutional review board (IRB) of the University of Turin. All patient data were securely stored within the hospital’s database and subsequently archived within an internal computerized system to ensure confidentiality and data integrity. Patients included in this study met specific inclusion criteria: female, aged 18 to 55 years at the time of initiation of biologic therapy, and had a confirmed clinical diagnosis of moderate-to-severe psoriasis. The severity of psoriasis was defined according to the current clinical guidelines and was characterized by a Psoriasis Area and Severity Index (PASI) score greater than 10 or a body surface area (BSA) involvement exceeding 10%. Additionally, a Dermatology Life Quality Index (DLQI) score greater than 10 was required for inclusion. Other criteria that could be used to elevate a patient’s disease classification from mild to moderate-to-severe included major involvement of visible areas, the scalp, or the genitals; onycholysis or onychodystrophy affecting at least two fingernails; significant pruritus leading to scratching; and the presence of recalcitrant plaques [17]. Patients with incomplete medical records or those who did not meet the defined inclusion criteria were excluded from this study.

This study evaluated the clinical outcomes of patients treated with six different IL-23 or IL-17 inhibitors, administered according to the approved regimens at the time of this study. The prescription of drugs was made in accordance with the regional health system’s prescribing guidelines and hospital policies. The specific biological agents and their dosing schedules were as follows:

- Ixekizumab: 80 mg subcutaneous injection every 4 weeks after initial induction.
- Secukinumab: 150 mg, administered as two subcutaneous injections every 4 weeks after induction.
- Brodalumab: 210 mg subcutaneous injection every 2 weeks.
- Guselkumab: 100 mg subcutaneous injection every 8 weeks.
- Risankizumab: 75 mg, administered as two subcutaneous injections every 12 weeks (150 mg formulation was not available during the study period).
- Tildrakizumab: 100 mg subcutaneous injection every 12 weeks.

The primary objectives of this study were to assess the effectiveness of these biologic treatments at 16 weeks (W), 28 weeks, and 52 weeks, as determined by achieving PASI 100 (complete clearance), PASI 90 (90% clearance), and PASI ≤ 3 (minimal disease). Secondary objectives included a comparative analysis of the effectiveness between IL-23 inhibitors and IL-17 inhibitors at the same time points, as well as an individual comparison of each treatment’s effectiveness using the same PASI endpoints. Data were extracted from the hospital’s comprehensive database, including demographic information, baseline disease characteristics, treatment history, and clinical outcomes. Continuous variables were reported as mean ± standard deviation (SD) or median with range, depending on the distribution assessed by the Shapiro–Wilk test. Categorical variables were presented as absolute and relative frequencies, with percentages calculated based on the number of non-missing values. The statistical significance was set at α = 0.05 for all tests, and all analyses were performed using Stata/SE v.18 software (StataCorp, College Station, TX, USA). Two-sided *p*-values were calculated for hypothesis testing, ensuring robustness in the results’ interpretation.

## 3. Results

As of June 2023, a total of 172 women aged between 20 and 55 years were identified as receiving treatment with IL-17 and IL-23 inhibitors for moderate to severe PSO between 1 January 2017 and 1 June 2022. To prevent pregnancy during treatment, all patients adhered to a dual contraception regimen. All patients were followed clinically for a minimum of 52 weeks to assess treatment effectiveness and safety. During the time of observation, no pregnancies were reported. The average age of these patients was 40.39 years. The average age of onset of PSO among these women was 22 years (SD 12.29). The average Body Mass Index (BMI) of the patients was 24.53 (SD 6.01). Within this group, 22 patients were classified as obese, highlighting a significant subset of the cohort with elevated BMI levels. Additionally, 26 patients reported cardiovascular comorbidities, and 3 patients had a diagnosis of diabetes. The majority of the patients (86%) experienced psoriasis involvement at difficult-to-treat sites.

Regarding the specific treatments received, 47.09% of the patients were administered IL-17 inhibitors, while 52.91% were treated with IL-23 inhibitors. Notably, 64.53% of the women (111 patients) were biologic-naïve prior to receiving these therapies. Among the treatment breakdown, 55 patients (33.14%) received risankizumab, 34 patients (19.77%) were treated with secukinumab, 24 patients (13.95%) received brodalumab, 23 patients (13.37%) were treated with ixekizumab, 18 patients (10.47%) received guselkumab, and 16 patients (9.3%) were treated with tildrakizumab.

At the initiation of treatment, the mean PASI score was 21.78 (SD 30.08), indicating moderate to severe disease activity across the cohort. By week 16 of treatment, the mean PASI score had significantly decreased to 3.36 (SD 9.1), reflecting a marked improvement in disease severity. This trend of improvement in PASI scores was consistently observed at subsequent time points throughout the follow-up period, demonstrating the sustained effectiveness of IL-17 and IL-23 inhibitor therapies in this patient group (Table 1).

**Figure 1:**
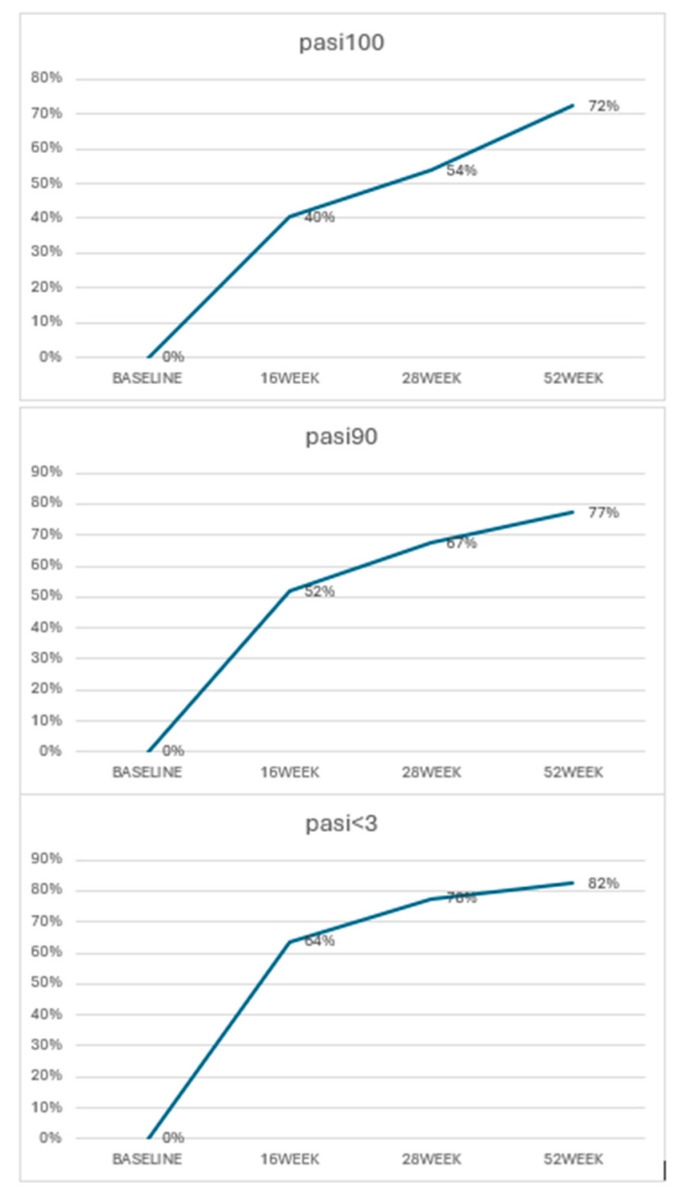
PASI achievements at different time points.

Overall, treatment outcomes showed significant improvement in psoriasis severity among the patient cohort. At week 16, 40% of patients achieved PASI 100, indicating complete clearance of skin lesions. This response rate increased over time, with 54% of patients achieving PASI 100 at week 28 and 72% at week 52. For those reaching PASI 90, the results were similar: 52% of patients reached this milestone at week 16, 67% at week 28, and 77% at week 52. Furthermore, 64% of patients achieved a PASI score of less than 3 at week 16, and this rate further increased to 78% at week 28 and 86% at week 52 (Figure 1).

A comparison of the effectiveness between IL-17 and IL-23 inhibitors at week 16 showed a statistically significant difference in achieving PASI 100. Specifically, 41 patients (24%) treated with IL-17 inhibitors reached PASI 100, compared to 27 patients (16%) treated with IL-23 inhibitors (*p* = 0.004). However, at weeks 28 and 52, there was no statistically significant difference between the two treatment groups, indicating that while IL-17 inhibitors may offer a quicker response, the long-term outcomes are comparable. The same pattern was observed for PASI 90 at weeks 16, 28, and 52, suggesting similar trends in effectiveness between the two types of inhibitors over time. No statistically significant differences were observed between the two groups for achieving PASI scores below 3 at any time point.

**Figure 2:**
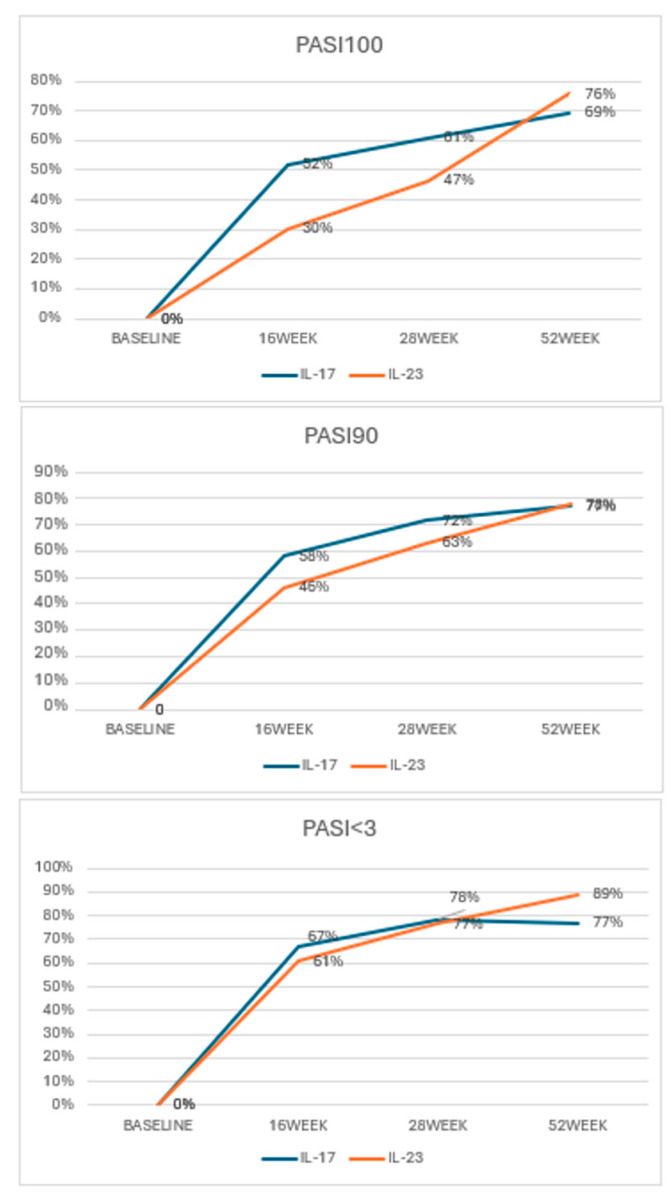
PASI achievements according to the different biologic classes.

Specifically, at week 16, 52% of patients on IL-17 reached PASI 100, compared to 30% of those on IL-23. By week 28, the rates increased to 61% for IL-17 and 47% for IL-23. At the end of the 52-week period, 76% of IL-17-treated patients achieved complete clearance, compared to 69% in the IL-23 group. The percentage of patients achieving a 90% improvement was slightly higher in the IL-17 group throughout this study. At week 16, 58% of IL-17-treated patients achieved PASI 90, compared to 46% of those treated with IL-23. By week 28, IL-17 further improved to 72%, while IL-23 reached 63%. At week 52, 77, and 78% of patients achieved PASI 90. IL-17 and IL-23 demonstrated similar effectiveness in reducing disease severity to PASI < 3. At week 16, 67% of patients treated with IL-17 achieved PASI < 3, closely followed by 61% of those on IL-23. At week 28, the rates were nearly identical, with 78% for IL-17 and 77% for IL-23. Notably, by week 52, IL-23 slightly outperformed IL-17, with 89% of patients reaching PASI < 3 compared to 77% in the IL-17 group. Overall, IL-17 inhibitors demonstrated a more rapid onset of response, yet by the 52-week mark, IL-23 inhibitors demonstrated comparable results, particularly in reducing disease severity to PASI < 3 (Figure 2).

According to different biologic treatments, the mean PASI (SD) at baseline ranged from 17.68 (5.59) with secukinumab to 35.17 (50.44) with guselkumab. At 16 weeks, brodalumab had a mean PASI of 2.39 (3.13), guselkumab 9.06 (25.27), ixekizumab 2.95 (4.42), risankizumab 3.25 (4.9), secukinumab 1.71 (2.33), and tildrakizumab 2.8 (3.23). At 28 weeks, the lowest mean PASI was observed with risankizumab at 1.13 (1.62), followed by ixekizumab at 1.67 (2.97) and brodalumab at 3.95 (7.4). By 52 weeks, the lowest mean PASI was observed with guselkumab at 0.92 (1.83) and risankizumab at 1.16 (5). Secukinumab and tildrakizumab showed mean PASI scores of 1.4 (3.06) and 2.13 (3), respectively.

The PASI 100 response showed that at 16 weeks, brodalumab achieved a response rate of 43%, guselkumab 39%, ixekizumab 59%, risankizumab 29%, secukinumab 53%, and tildrakizumab 27%. At 28 weeks, response rates increased, with brodalumab at 60%, guselkumab at 50%, ixekizumab at 62%, risankizumab at 53%, secukinumab at 61%, and tildrakizumab at 21%. At 52 weeks, brodalumab reached an 80% response rate, guselkumab 67%, ixekizumab 65%, risankizumab 88%, secukinumab 70%, and tildrakizumab 50%.

PASI 90 response rates were 43% for brodalumab, 50% for guselkumab, 64% for ixekizumab, 48% for risankizumab, 59% for secukinumab, and 33% for tildrakizumab at 16 weeks. At 28 weeks, the response rates were 70% for brodalumab, 57% for guselkumab, 71% for ixekizumab, 73% for risankizumab, 73% for secukinumab, and 36% for tildrakizumab. By 52 weeks, the response rates were 80% for brodalumab, 67% for guselkumab, 76% for ixekizumab, 88% for risankizumab, 77% for secukinumab, and 63% for tildrakizumab.

The PASI < 3 response, representing minimal disease activity, showed brodalumab at 65%, guselkumab at 67%, ixekizumab at 64%, risankizumab at 63%, secukinumab at 71%, and tildrakizumab at 47% at 16 weeks. At 28 weeks, the response rates increased to 85% for brodalumab, 71% for guselkumab, 76% for ixekizumab, 80% for risankizumab, 76% for secukinumab, and 71% for tildrakizumab. At 52 weeks, brodalumab had an 80% response rate, guselkumab 83%, ixekizumab 76%, risankizumab 100%, secukinumab 77%, and tildrakizumab 63%.

**Figure 3:**
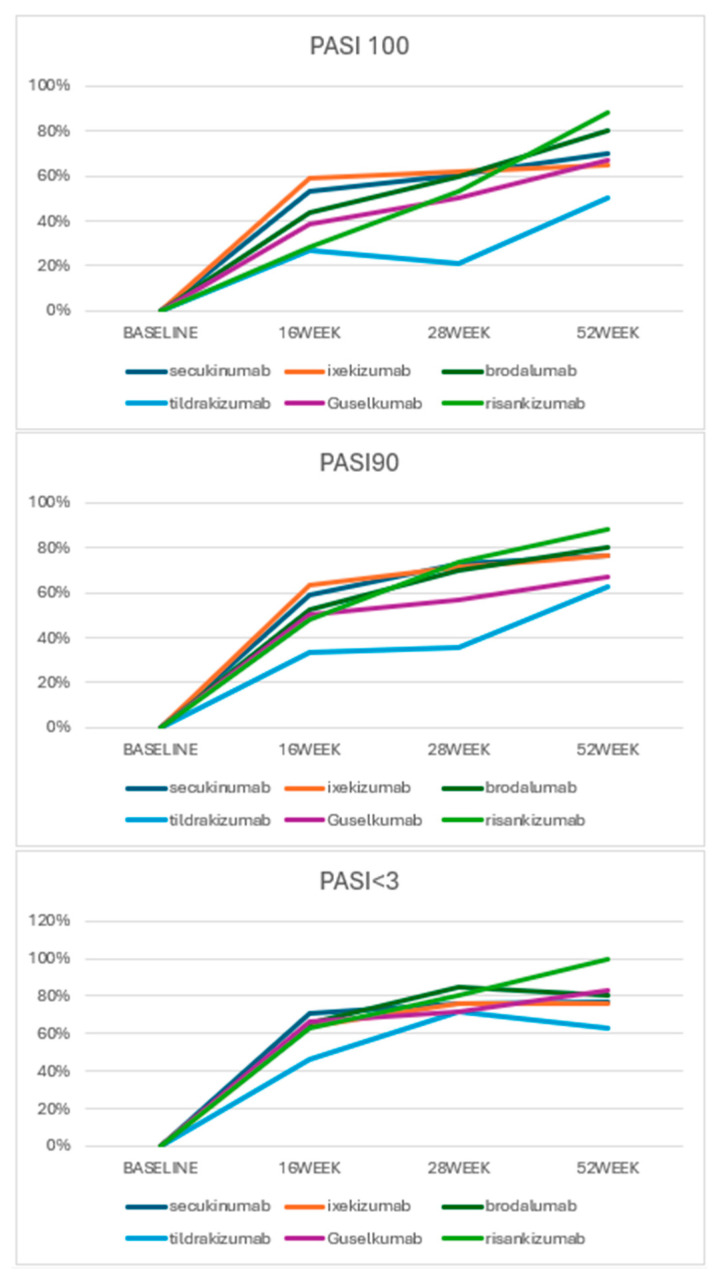
PASI achievements according to the different biologic drugs.

Overall, no statistically significant differences were noted in PASI achievement rates at any evaluated time points. However, tildrakizumab showed a slightly slower response compared to the other drugs, though this difference was not statistically significant (Table 2, Figure 3).

Throughout this study, 46 patients (33%) discontinued treatment. Adverse effects were reported by 32 women (19%). The reported side effects in the study population varied in frequency, with rhinitis being the most common (*n* = 6). Headache was the next most frequently reported side effect (*n* = 5), followed by fatigue (*n* = 3) and injection site pain (*n* = 3). Nausea and candidiasis were reported twice each (*n* = 2), as were acne (*n* = 2) and diarrhea (*n* = 2). Several other side effects were noted only once, including arthralgia (*n* = 1), herpes zoster (*n* = 1), herpes simplex (*n* = 1), skin abscess (*n* = 1), otitis (*n* = 1), defluvium (*n* = 1), gastroesophageal reflux disease (*n* = 1), lack of appetite (*n* = 1), and heart pounding (*n* = 1). Overall, the majority of side effects were mild and infrequent, with no serious or life-threatening events. Overall, while adverse effects were noted, their generally mild nature and the lack of serious or life-threatening events confirmed the favorable safety profile of the biologic treatments.

## 4. Discussion

Psoriasis is a chronic inflammatory disease that, while primarily affecting the skin, also has significant systemic implications extending to various aspects of women’s health, including fertility. The relationship between PSO and fertility is complex and somewhat controversial. Some studies suggest that PSO might directly or indirectly influence fertility in women of childbearing age. Ayanoglu et al. and Mathyk et al. have highlighted a reduced ovarian reserve in women with PSO, suggesting a potential impact on fertility [30,31]. This reduced ovarian reserve could be a critical factor contributing to fertility challenges in these women. Furthermore, PSO is often associated with cardiovascular and metabolic comorbidities, such as obesity, diabetes, and hypertension, which could further exacerbate fertility issues. These comorbidities are known to influence reproductive health, and their prevalence in women with PSO could partially explain the observed fertility challenges. The exact mechanisms by which PSO and its associated comorbidities affect fertility are still under investigation, but it is likely that the systemic nature of PSO plays a crucial role in this context [32].

In this scenario, our study has provided valuable insights into the management of PSO in women of childbearing age, particularly regarding the use of biologic therapies.

The study limitations include its retrospective and real-life design, the absence of a control group, and the restriction to case analysis over a limited time period. Additionally, this study did not assess quality of life measures, nor did it examine the ovarian reserve before and after induction and at the end of treatment.

Biologic drugs have emerged as a cornerstone in the treatment of moderate to severe PSO, offering significant improvements in skin clearance and overall disease management [17]. In the literature, there are few data on the safety of these drugs in childbearing age and pregnancy. With regard to interleukin-17 inhibitors, such as ixekizumab, brodalumab, and secukinumab in pregnancy, data are limited. However, animal studies have shown no adverse effects of ixekizumab and secukinumab on the fetus [33,34]. There are no studies on the efficacy and safety of brodalumab in the treatment of pregnant or lactating women with psoriasis; therefore, risks and benefits should be analyzed before using brodalumab as a form of treatment for this category of patients [35].

Ustekinumab, a monoclonal antibody that inhibits the action of interleukins IL-12, IL-13, and IL-23, is classified by the FDA as category B [11]. However, data on the safety of ustekinumab in pregnancy are limited. There are many cases in the literature of the birth of healthy babies, but also cases of miscarriages in pregnant women [36,37]. Concerning interleukin-23 inhibitors, specifically the IL-23A inhibitor risankizumab, an open-label phase 2 extension study investigating its long-term safety and efficacy reported three pregnancies: two without complications or abnormalities and one with fetal defects, which resulted in a surgical abortion [38].

Our findings indicate that anti-IL therapies are not only effective but also safe for women of childbearing age, with minimal side effects. One notable aspect of our study is the comparison of different classes of biologic drugs, particularly IL-17 and IL-23 inhibitors. We observed that IL-17 inhibitors tend to have a more rapid onset of action, achieving skin improvements faster than IL-23 inhibitors. This observation aligns with the existing literature, where IL-17 inhibitors have been shown to offer a quicker response in terms of skin clearance [39]. However, over time, both IL-17 and IL-23 inhibitors demonstrated similar efficacy in managing PSO. This is consistent with the findings of Mastorino et al., who reported that while IL-17 inhibitors provided a faster response by week 16, IL-23 inhibitors showed progressive improvement during follow-up visits [40]. In our study, we also observed that tildrakizumab, an IL-23 inhibitor, showed a slower response in the first year of therapy but eventually reached a comparable efficacy to IL-17 inhibitors, such as brodalumab and risankizumab. This suggests that while initial response rates may vary, the long-term outcomes of these biologic therapies are similar, providing effective disease control for women of childbearing age. The differences in responses between these drug classes did not reach statistical significance, underscoring the importance of personalized treatment approaches based on individual patient characteristics and preferences.

The safety of biologic therapies is a crucial consideration for women with PSO who are of childbearing age. Recent research by Sanchez-Gracia et al. has provided reassuring data, indicating that exposure to biologic therapies during pregnancy and conception does not appear to increase the risk of miscarriage, abortion, or congenital malformations. The reported rates of these adverse outcomes were similar to those observed in the general population, underscoring the safety of biologic treatments during this critical period [41,42]. These data align with the multicentric observational PSOLAR study, which evaluated pregnancy outcomes in women with psoriasis who received biological drugs during pregnancy or the prenatal period, showing rates of miscarriage, neonatal problems, and congenital malformations similar to those in the general population [43].

In conclusion, the decision to initiate, maintain, or discontinue biologic therapies in women of childbearing age requires careful evaluation of the risks and benefits on a case-by-case basis. Important factors include the severity of the disease, quality of life, pregnancy planning, patient preferences, and the potential for transplacental transfer of the specific biologic agent. Counseling in these cases is crucial, as women with PSO who do not plan pregnancy may consider anti-IL treatments over less effective medications. Current evidence suggests that in many cases of moderate to severe PSO, the benefits of biologic therapy outweigh the potential risks. However, ongoing monitoring and caution are warranted, as research in this area is still evolving. Ensuring informed decision-making about treatment options is key to balancing effective disease management with the desire for a healthy pregnancy and child. A strong patient–doctor relationship plays a pivotal role in this context. Open communication allows for tailored discussions around planning pregnancy, adjusting treatment regimens, and managing PSO effectively. Physicians should foster a supportive environment where women feel comfortable discussing their reproductive goals, enabling shared decision-making that considers the patient’s lifestyle, treatment preferences, and future family planning. This collaborative approach is essential in empowering women with PSO to navigate their treatment choices confidently, ensuring that their journey towards managing their disease is aligned with their reproductive aspirations.

## Figures and Tables

**Table 1:** Study population and PASI score according to different biologics and time points.

	Baseline	16 Weeks	28 Weeks	52 Weeks
Mean PASI (SD)	21.78 (30.08)	3.36 (9.1)	2.03 (4.31)	1.36 (3.49)
PASI 100	0	40%	54%	72%
PASI 90	0	52%	67%	77%
PASI < 3	0	64%	78%	82%
Mean PASI (SD) IL17	20.96 (21.86)	2.25 (3.26)	2.31 (4.6)	1.44 (3)
Mean PASI (SD) IL23	22.52 (35.95)	4.35 (12.07)	1.74 (3.99)	1.27 (4.01)
*p*-value	0.736	0.137	0.423	0.806
PASI 100 IL17		52%	61%	69%
PASI 100 IL23		30%	47%	76%
*p*-value		0.04	0.083	0.488
PASI 90 IL17		58%	72%	77%
PASI 90 IL23		46%	63%	78%
*p*-value		0.115	0.266	0.92
PASI < 3 IL17		67%	78%	77%
PASI < 3 IL23		61%	77%	89%
*p*-value		0.388	0.809	0.122

**Table 2:** PASI scores according to different biologics and time points.

	Treatment	Baseline	16 Weeks	28 Weeks	52 Weeks
meanPASI (SD)	Brodalumab	28.63 (38.64)	2.39 (3.13)	3.95 (7.4)	2.4 (5.37)
	Guselkumab	35.17 (50.44)	9.06 (25.27)	3.5 (8.4)	0.92 (1.83)
	Ixekizumab	17.83 (6.28)	2.95 (4.42)	1.67 (2.97)	1.24 (2.05)
	Risankizumab	19.74 (31.28)	3.25 (4.9)	1.13 (1.62)	1.16 (5)
	Secukinumab	17.68 (5.59)	1.71 (2.33)	1.73 (2.82)	1.4 (3.06)
	Tildrakizumab	18.19 (31.29)	2.8 (3.23)	1.93 (2.06)	2.13 (3)
*p*-value		0.276	0.127	0.161	0.954
PASI 100	Brodalumab		43%	60%	80%
	Guselkumab		39%	50%	67%
	Ixekizumab		59%	62%	65%
	Risankizumab		29%	53%	88%
	Secukinumab		53%	61%	70%
	Tildrakizumab		27%	21%	50%
*p*-value			0.077	0.188	0.311
PASI 90	Brodalumab		43%	70%	80%
	Guselkumab		50%	57%	67%
	Ixekizumab		64%	71%	76%
	Risankizumab		48%	73%	88%
	Secukinumab		59%	73%	77%
	Tildrakizumab		33%	36%	63%
*p*-value			0.512	0.134	0.633
PASI < 3	Brodalumab		65%	85%	80%
	Guselkumab		67%	71%	83%
	Ixekizumab		64%	76%	76%
	Risankizumab		63%	80%	100%
	Secukinumab		71%	76%	77%
	Tildrakizumab		47%	71%	63%
*p*-value			0.745	0.916	0.123

## Data Availability

Data are available upon reasonable request to the corresponding author.

## References

[1] World Health Organization (2016). Global Report on Psoriasis.

[2] Griffiths C.E.M., Armstrong A.W., Gudjonsson J.E., Barker J.N.W.N. (2021). Psoriasis. Lancet.

[3] Levine D., Gottlieb A. (2009). Evaluation and management of psoriasis: An internist’s guide. Med. Clin. N. Am..

[4] Horn E.J., Chambers C.D., Menter A., Kimball A.B., International Psoriasis Council (2009). Pregnancy outcomes in psoriasis: Why do we know so little?. J. Am. Acad. Dermatol..

[5] Nasca M.R., Giuffrida G., Micali G. (2021). The influence of pregnancy on the clinical evolution and prognosis of pre-existing inflammatory and autoimmune skin disorders and their management. Dermatology.

[6] Huang Y.H., Yee N.C., Chiou M.J., Kuo C.F. (2021). Fetal-neonatal and maternal outcomes in women with psoriasis vulgaris: A nationwide population-based registry linkage study in Taiwan. J. Dermatol..

[7] Guven M.A., Coskun A., Ertas I.E., Aral M., Zencirci B., Oksuz H. (2009). Association of maternal serum CRP, IL-6, TNF-alpha, homocysteine, folic acid and vitamin B12 levels with the severity of preeclampsia and fetal birth weight. Hypertens Pregnancy.

[8] Roccuzzo G., Gherardi E., Maio M., Malagoli P., Marzano A.V., Parodi A., Pimpinelli N., Spagnolo F., Di Giacomo A.M., Quaglino P. (2024). Immunotherapy in cutaneous melanoma and biologics in psoriatic disease: Similarities and differences from a clinical multidisciplinary perspective. Expert Opin. Biol. Ther..

[9] Armstrong A.W., Puig L., Joshi A., Skup M., Williams D., Li J., Betts K.A., Augustin M. (2020). Comparison of Biologics and Oral Treatments for Plaque Psoriasis: A Meta-analysis. JAMA Dermatol..

[10] Paziana K., del Monaco M., Cardonick E., Moritz M., Keller M., Smith B., Coscia L., Armenti V. (2013). Ciclosporin use during pregnancy. Drug Saf..

[11] Pomeranz M.K., Strober B.E., Post T.W. (2022). Management of psoriasis in pregnancy. UpToDate.

[12] Geiger J.M., Baudin M., Saurat J.H. (1994). Teratogenic risk with etretinate and acitretin treatment. Dermatology.

[13] Porter M.L., Lockwood S.J., Kimball A.B. (2017). Update on biologic safety for patients with psoriasis during pregnancy. Int. J. Womens Dermatol..

[14] Lebwohl M., Van Vorhees A.S., Siegel M., Shankle L., Pisenti L., Yassine M. (2018). A comprehensive survey assessing the family planning needs of women with psoriasis. Acta Derm. Venereol..

[15] Smith C.H., Jabbar-Lopez Z.K., Yiu Z.Z., Bale T., Burden A.D., Coates L.C., Cruickshank M., Hadoke T., MacMahon E., Murphy R. (2017). British Association of Dermatologists guidelines for biologic therapy for psoriasis 2017. Br. J. Dermatol..

[16] Kane S.V., Acquah L.A. (2009). Placental transport of immunoglobulins: A clinical review for gastroenterologists who prescribe therapeutic monoclonal antibodies to women during conception and pregnancy. Am. J. Gastroenterol..

[17] Nast A., Smith C., Spuls P.I., Avila Valle G., Bata-Csörgö Z., Boonen H., De Jong E., Garcia-Doval I., Gisondi P., Kaur-Knudsen D. (2021). EuroGuiDerm Guideline on the systemic treatment of Psoriasis vulgaris—Part 2: Specific clinical and comorbid situations. J. Eur. Acad. Dermatol. Venereol. JEADV.

[18] Johansen C.B., Jimenez-Solem E., Haerskjold A., Sand F.L., Thomsen S.F. (2018). The Use and Safety of TNF Inhibitors during Pregnancy in Women with Psoriasis: A Review. Int. J. Mol. Sci..

[19] Nast A., Amelunxen L., Augustin M., Boehncke W.H., Dressler C., Gaskins M., Härle P., Hoffstadt B., Klaus J., Koza J. (2018). S3 Guideline for the treatment of psoriasis vulgaris, update—Short version part 1—Systemic treatment. J. Der Dtsch. Dermatol. Ges..

[20] Carter J.D., Valeriano J., Vasey F.B. (2006). Tumor necrosis factor-alpha inhibition and VATER association: A causal relationship. J. Rheumatol..

[21] Rademaker M., Agnew K., Andrews M., Armour K., Baker C., Foley P., Frew J., Gebauer K., Gupta M., Kennedy D. (2018). Psoriasis in those planning a family, pregnant or breast-feeding. The Australasian Psoriasis Collaboration. Australas. J. Dermatol..

[22] Roccuzzo G., Mastorino L., Susca S., Cariti C., Passerini S.G., Sciamarrelli N., Borriello S., Macagno N., Sliquini N., Avallone G. (2023). Drug survival and efficacy of anti-interleukin 23 biologics in psoriasis: A comparative study on different agents. Clin. Exp. Dermatol..

[23] Mastorino L., Susca S., Cariti C., Sliquini N., Verrone A., Stroppiana E., Ortoncelli M., Dapavo P., Ribero S., Quaglino P. (2023). Efficacy of anti-IL-23 and anti-IL-17 after adalimumab failure in psoriatic patients. J. Eur. Acad. Dermatol. Venereol. JEADV.

[24] Sammaritano L.R., Bermas B.L., Chakravarty E.E., Chambers C., Clowse M.E.B., Lockshin M.D., Marder W., Guyatt G., Branch D.W., Buyon J. (2020). 2020 American College of Rheumatology Guideline for the Management of Reproductive Health in Rheumatic and Musculoskeletal Diseases. Arthritis Care Res..

[25] European Medicines Agency Skyrizi: Summary of Product Characteristics. https://www.ema.europa.eu/en/documents/product-information/skyrizi-epar-product-information_en.pdf.

[26] Bucur Ș., Savu A.P., Stănescu A.M.A., Șerban E.D., Nicolescu A.C., Constantin T., Bobircă A., Constantin M.M. (2022). Oversight and Management of Women with Psoriasis in Childbearing Age. Medicina.

[27] Owczarek W., Walecka I., Lesiak A., Czajkowski R., Reich A., Zerda I., Narbutt J. (2020). The use of biological drugs in psoriasis patients prior to pregnancy, during pregnancy and lactation: A review of current clinical guidelines. Postepy Dermatol. Alergol..

[28] Pottinger E., Woolf R.T., Exton L.S., Burden A.D., Nelson-Piercy C., Smith C.H. (2018). Exposure to biological therapies during conception and pregnancy: A systematic review. Br. J. Dermatol..

[29] Salaffi F., Carotti M., Gasparini S., Intorcia M., Grassi W. (2009). The health-related quality of life in rheumatoid arthritis, ankylosing spondylitis, and psoriatic arthritis: A comparison with a selected sample of healthy people. Health Qual. Life Outcomes.

[30] Tuğrul Ayanoğlu B., Özdemir E.D., Türkoğlu O., Alhan A. (2018). Diminished ovarian reserve in patients with psoriasis. Taiwan J. Obstet. Gynecol..

[31] Aydogan Mathyk B., Aslan Cetin B., Bilici S., Fasse J., Avci P. (2019). Evaluation of ovarian reserve in women with psoriasis. Gynecol. Endocrinol..

[32] De Simone C., Caldarola G., Moretta G., Piscitelli L., Ricceri F., Prignano F. (2019). Moderate-to-severe psoriasis and pregnancy: Impact on fertility, pregnancy outcome and treatment perspectives. G. Ital. Dermatol. Venereol..

[33] European Medicines Agency Taltz: Summary of Product Characteristics. https://www.ema.europa.eu/en/documents/product-information/taltz-epar-product-information_en.pdf.

[34] European Medicines Agency Cosentyx: Summary of Product Characteristics. https://www.ema.europa.eu/en/documents/product-information/cosentyx-epar-product-information_en.pdf.

[35] Golbari N.M., Basehore B.M., Zito P.M. (2021). Brodalumab. StatPearls.

[36] Sheeran C., Nicolopoulos J. (2014). Pregnancy outcomes of two patients exposed to ustekinumab in the first trimester. Australas. J. Dermatol..

[37] Fotiadou C., Lazaridou E., Sotiriou E., Ioannides D. (2012). Spontaneous abortion during ustekinumab therapy. J. Dermatol. Case Rep..

[38] Ferrante M., Feagan B.G., Panés J., Baert F., Louis E., Dewit O., Kaser A., Duan W.R., Pang Y., Lee W.-J. (2021). Long-Term Safety and Efficacy of Risankizumab Treatment in Patients with Crohn’s Disease: Results from the Phase 2 Open-Label Extension Study. J. Crohn’s Colitis.

[39] Blauvelt A., Papp K., Gottlieb A., Jarell A., Reich K., Maari C., Gordon K.B., Ferris L.K., Langley R.G., Tada Y. (2020). A head-to-head comparison of ixekizumab vs. guselkumab in patients with moderate-to-severe plaque psoriasis: 12-week efficacy, safety and speed of response from a randomized, double-blinded trial. Br. J. Dermatol..

[40] Mastorino L., Dapavo P., Susca S., Cariti C., Siliquini N., Verrone A., Stroppiana E., Ortoncelli M., Quaglino P., Ribero S. (2024). Drug survival and clinical effectiveness of secukinumab, ixekizumab, brodalumab, guselkumab, risankizumab, tildrakizumab for psoriasis treatment. J. Der Dtsch. Dermatol. Ges..

[41] Sánchez-García V., Hernández-Quiles R., de-Miguel-Balsa E., Giménez-Richarte Á., Ramos-Rincón J.M., Belinchón-Romero I. (2023). Exposure to biologic therapy before and during pregnancy in patients with psoriasis: Systematic review and meta-analysis. J. Eur. Acad. Dermatol. Venereol..

[42] Yeung J., Gooderham M.J., Grewal P., Hong C.H., Lansang P., Papp K.A., Poulin Y., Turchin I., Vender R. (2020). Management of Plaque Psoriasis with Biologic Therapies in Women of Child-Bearing Potential Consensus Paper. J. Cutan. Med. Surg..

[43] Kimball A.B., Guenther L., Kalia S., De Jong E.M., Lafferty K.P., Chen D.Y., Langholff W., Shear N.H. (2021). Pregnancy Outcomes in Women with Moderate-to-Severe Psoriasis from the Psoriasis Longitudinal Assessment and Registry (PSOLAR). JAMA Dermatol..

